# Significance of Interleukin-33 and Its Related Cytokines in Patients with Breast Cancers

**DOI:** 10.3389/fimmu.2014.00141

**Published:** 2014-04-07

**Authors:** Jing Liu, Jia-Xin Shen, Jia-Lin Hu, Wen-He Huang, Guo-Jun Zhang

**Affiliations:** ^1^Cancer Research Center, Shantou University Medical College, Shantou, China; ^2^Breast Center, Cancer Hospital of Shantou University Medical College, Shantou, China

**Keywords:** interleukin-33, ST2/ST2L, breast cancer, cytokines, immunosuppression

## Abstract

Interleukin-33 (IL-33) is a recently identified cytokine, an important member of the interleukin-1 family. IL-33 binds to its receptor ST2 to induce type 2 cytokines and exert both pro-inflammatory and protective functions in host defense and disease. Murine breast carcinoma models suggest disruption of ST2 signaling may enhance the anti-tumor immune response, suggesting IL-33 impedes anti-tumor immunity. However, the role of IL-33 in patients with breast cancers (BC) is not elucidated. We detected the expression of IL-33 in tumor tissue, and IL-33 and its related cytokines in serum from BC patients. Using Luminex and immunohistochemistry methods, we found that serum levels of IL-33 were nearly twofold higher in patients with BC, compared to patients with benign breast diseases. In cancer tissues, expression of IL-33 was higher than matched normal breast tissues from the same patients, and was also associated with a well-differentiated phenotype, HER2 overexpression, more lymph nodes involvement, and a family history of malignant carcinoma. These results suggest that IL-33 may play an important role in the progress of BC and may be a useful biomarker for predicting the progress and metastasis of BC.

## Introduction

Breast cancer (BC) is the second leading cause of cancer-related deaths amongst women in the United States, and morbidity and mortality of this disease increases each year (1). Although standard multi-modality treatment has improved the overall outcome and quality of life for patients with BC, identification of new prognostic markers, therapeutic targets, and new therapeutic approaches are needed. Recent insights into the cancer development mechanisms have revealed that immune system functionally regulates development and progression of epithelial malignancies and tumor-infiltrating leukocytes may be causal players in cancer development (2).

The interleukin-1 (IL-1) family is a growing group of cytokines, consisting of at least 11 members, and the balance between pro- and anti-inflammatory cytokines is crucial in the pathogenesis of many human diseases (3). Interleukin-33 (IL-33) is an important member of the IL-1 family, and in humans is expressed predominantly in skin, lung, adipocytes, and synovial fibroblasts (4). IL-33 is an endogenous ligand for the ST2/T1 receptor, and depending on the cellular and cytokine context, participates in many immune diseases with dual, pro-inflammatory, or protective roles. IL-33 induces T cells to produce IL-4, IL-5, and IL-13, and potently induces pro-inflammatory cytokines and chemokines through a Th2-dependent pathway, and also promotes Th1-type responses (5). IL-33 is involved in the pathogenesis of immune diseases, such as rheumatoid arthritis and atopic dermatitis, and may reflect the degree of inflammation in patients with immune diseases (6, 7). Deletion of IL-33/ST2 function enhances cytotoxicity of NK cells and increases levels of TNF-α, IFN-γ, and IL-17, and systemic pro-inflammatory cytokines, leading to attenuated tumor growth (8).

Recent studies demonstrated that high serum levels of soluble ST2 (sST2) are a worse prognostic factor in hepatocellular carcinoma (9), and that serum IL-33 is a diagnostic and prognostic marker in non-small cell lung cancer, independent of the therapeutic intervention (10). In studies on mouse mammary carcinoma, the IL-33/ST2 pathway promotes BC progression and metastasis through increased intratumoral accumulation of immunosuppressive cells and by diminishing innate anti-tumor immunity (11). Conversely, Gao et al. reported that transgenic expression of IL-33 may activate CD8(+) T cells and NK cells, and inhibit tumor growth and metastasis in B16 melanoma and Lewis lung carcinoma metastatic models (12). Thus, the data on the role of IL-33 in cancer progression was limited, and in particular, the function mediated by IL-33 in human BC is under-investigated. In this study, we aim to determine the serum level and to detect the expression of IL-33 in human tissues in patients with breast carcinoma, using Luminex-based measurements and immunohistochemistry, to further explore the role of IL-33 in anti-tumor immunity in BC.

## Materials and Methods

### Patients

This study was approved by the Ethics Committee of Shantou University Medical College and conducted according to the principles in the Declaration of Helsinki (13).

Blood samples were drawn from 64 patients with BC and 10 patients with benign breast diseases (BBD) as controls, who visited the Shantou University Medical College Cancer Hospital Breast Center between April 2013 and July 2013. The mean age was 52 ± 11 years (25–80 years old) for patients with BC, and 41 ± 11 years (28–68 years old) for patients with BBD. All patients were pathologically diagnosed using specimens obtained either by core-needle biopsy or by surgery. The clinicopathological characters of the BC patients are summarized in Table 1.

**Table 1:** **Relationship of serum IL-33 levels with clinicopathological parameters of breast cancer patients**.

Variables	IL-33 level (pg/ml)			
	*N*	Mean	SD	P value
Age				
≤50 years	30	35.87	11.00	0.481
>50 years	34	38.21	14.78	
Menopausal status^a^				
No	33	36.19	11.33	0.992
Yes	30	36.52	12.26	
Size				
≤2 cm	26	36.50	11.52	0.760
>2 cm	38	37.53	14.21	
AJCC stage				
I + II	44	37.93	12.11	0.465
III	20	35.32	15.23	
Histological grade^b^				
1 + 2	30	36.51	13.58	0.915
3	23	36.14	10.64	
ER expression^c^				
Negative	26	32.79	10.84	0.033
Positive	37	39.92	13.97	
PR expression^c^				
Negative	32	36.76	16.28	0.896
Positive	31	37.20	9.17	
HER2 expression^c^				
No	46	37.82	14.28	0.409
Yes	17	34.71	9.48	
Ki-67 expression^c^				
Low	15	43.74	19.40	0.021
High	48	34.86	9.86	
Lymph node metastasis				
≤3	56	37.59	13.52	0.445
>3	8	33.78	9.67	
Family history				
No	58	36.00	11.69	0.034
Yes	6	47.81	21.29	

*^a^One case missing this information because the patient was male*.

*^b^Two cases with invasive lobular carcinomas, one case with an intraductal papillary carcinoma, and in eight cases, only biopsy samples were available without information on histological grade*.

*^c^One case was discharged after biopsy, without information on ER, PR, HER2, and Ki-67 expression*.

The central regions of tumors were collected from 29 BC patients, as well as microscopic normal tissues from either tumor-adjacent normal tissue (<1 cm) or normal tissue ≥5 cm away from the tumor margins, for which paraffin-embedded samples were available. The mean age was 53 ± 13 years (25–80 years old) for all enrolled patient. The clinicopathological characteristics of the 29 BC patients are summarized in Table 3.

### Assessment of serum cytokines

The levels of IL-33, IL-12, IL-13, IL-17, IFN-γ, and TNF-α in serum were measured based on a Luminex assay, using Milliplex™ MAP (Millipore, MA, USA) multiplex magnetic bead-based antibody detection kits according to the manufacturer’s protocols (14).

Blood from patients was collected and centrifuged for 10 min at room temperature. Serum was removed carefully and stored at −80°C until use. Twenty-five microliters of neat samples were added into each well of 96-well plate, and then 25 μl mixed beads were added to the samples. The plate was incubated with agitation on a plate shaker overnight at 4°C. Twenty-five microliters of anti-cytokine antibody was then added and incubated for 1 h at room temperature. Lastly, 25 μl streptavidin–phycoerythrin was added into each well containing the detection antibodies for 30 min at room temperature. Cytokines were quantified using a BioPlex 200 platform (BioRad, CA, USA).

### Immunohistochemistry

Surgical specimens of cancer tissues, adjacent tissues to tumors, and normal tissues collected from patients with BC were formalin-fixed, paraffin-embedded, and cut into four-micron-thick sections. Sections were deparaffinized by immersion in xylene, and rehydrated in a series of graded alcohols. Epitope retrieval and inactivation of endogenous peroxidase activity was achieved as described (15). Samples were incubated overnight at 4°C with anti-IL-33 (R&D Systems, Minneapolis, MN, USA), then visualized using 3,3′-diaminobenzidine tetrahydrochloride (DAB). Sections without primary antibody were used as negative controls. Counterstaining was carried out with hematoxylin, and sections were visualized and photographed under a bright-field microscope (Olympus, Tokyo, Japan). Immunohistochemical staining was mainly cytoplasmic, and the percentage of positive cells for IL-33 was calculated for analysis by counting at least 200 cells in five or more high power fields.

Estrogen receptor (ER) and progesterone receptor (PR) were interpreted as negative or positive, if equal to and less than 1% or more than 1% of tumor showed nuclear positivity, respectively. HER2 was interpreted as negative or over-expressed, if there was 0–1+ or 3+ membranous staining, respectively. If there was 2+ membranous staining, FISH was conducted to determine whether HER2 was over-expressed (16). A Ki-67 cut-off point of 15% was defined according to the experience of different pathologists as well as national and international recommendations at present (17).

### Statistical analysis

Serum levels and tissue expression are expressed as the mean ± standard deviation (SD). Differences between groups were analyzed using the Student t-test or the non-parametric Mann–Whitney test. All statistical differences were considered significant at the level of *p* < 0.05. All data were analyzed with SPSS 19.0 software for Windows.

## Results

### Serum levels of IL-33 and related cytokines in BBD and BC

**Figure 1:**
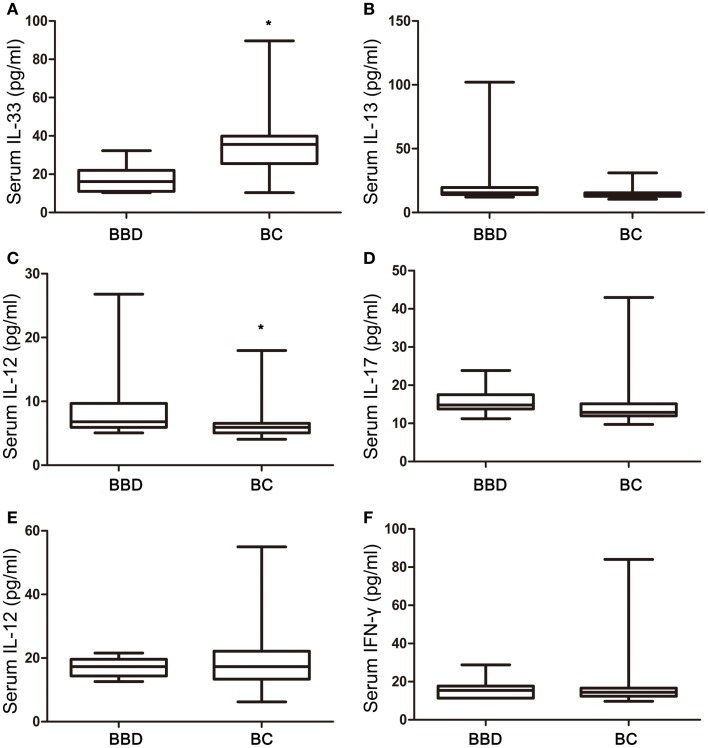
**Interleukin-33 and associated cytokine levels in benign breast diseases (BBD) and breast cancers (BC)**. **(A)** Serum IL-33 expression in BC was significant higher than that in BBD (p = 0.0008); **(B)** no significant difference between serum IL-13 expression in BC and BBD (p = 0.0608); **(C)** serum IL-12 expression in BBD was significant higher than that in BC (p = 0.0178); **(D)** no significant difference between serum IL-17 expression in BC and BBD (p = 0.0526); **(E)** no significant difference between serum TNF-α expression in BC and BBD (p = 0.9748); **(F)** no significant difference between serum IFN-γ expression in BC and BBD (p = 0.7693); *p < 0.05 by unpaired two tailed Student’s t-test.

Among six cytokines, the concentrations of IL-33 were nearly twofold higher in the BC group (34.49 ± 1.65 pg/ml) compared with BBD group (17.71 ± 2.60 pg/ml) (p = 0.0008), with a non-normal distribution (Figure 1A). Conversely, serum levels of IL-13, a Th2-associated cytokine, in the BC group (14.79 ± 0.45) were 40% lower than in the BBD group (24.92 ± 8.68 pg/ml) with borderline difference (*p* = 0.0608) (Figure 1B), and the concentrations of IL-12, a Th1-type cytokine, were 35% lower in the BC group (6.143 ± 0.25 pg/ml) than in the BBD group (9.39 ± 2.11 pg/ml) with statistical significance (p = 0.0178) (Figure 1C). The concentrations of other cytokines, IL-17, TNF-α, and IFN-γ, did not show any significant difference between the BBD and BC groups (p > 0.05, Figures 1D–F).

### Clinicopathological analysis of serum levels of IL-33 in breast carcinoma patients

In patients with ER-positive breast tumors, the serum levels of IL-33 were 39.92 ± 13.97 pg/ml, and were significantly higher than in patients with ER-negative tumors, which were 32.79 ± 10.84 pg/ml, p = 0.033 (Table 1). In patients who showed lower Ki-67 expression, the serum levels of IL-33 were higher than the high Ki-67-expressing group (43.74 ± 19.40 pg/ml vs. 34.86 ± 9.86 pg/ml, p = 0.021). The serum concentrations of IL-33 were significantly associated with family history of malignant tumors (p = 0.034). No correlation was observed between serum IL-33 levels and patient age, menopausal status, tumor size, AJCC stage, histological grade, lymph node status, PR, and HER2 expression.

We also analyzed the association of clinicopathological parameters with IL-33-related cytokines. The serum levels of INF-γ were associated with tumor size (p = 0.039), with INF-γ being higher in patients with tumors <2 cm in size (22.57 ± 2.63 pg/ml) than patients with tumors >2 cm (15.22 ± 5.13 pg/ml). In pre-menopausal patients, the serum levels of IL-17 were significantly higher (p = 0.048) than that in post-menopausal group (15.51 ± 6.29 vs. 13.13 ± 2.16 pg/ml), and serum levels of IL-17 were also significantly associated with AJCC stage (p = 0.049) and HER2 expression (p = 0.012). No statistical significance was found between the serum levels of TNF-α, IL-12, or IL-13 with clinicopathological parameters of BC patients (specific data not shown).

### IL-33 is higher in cancer tissues, compared with adjacent and normal tissues

**Figure 2:**
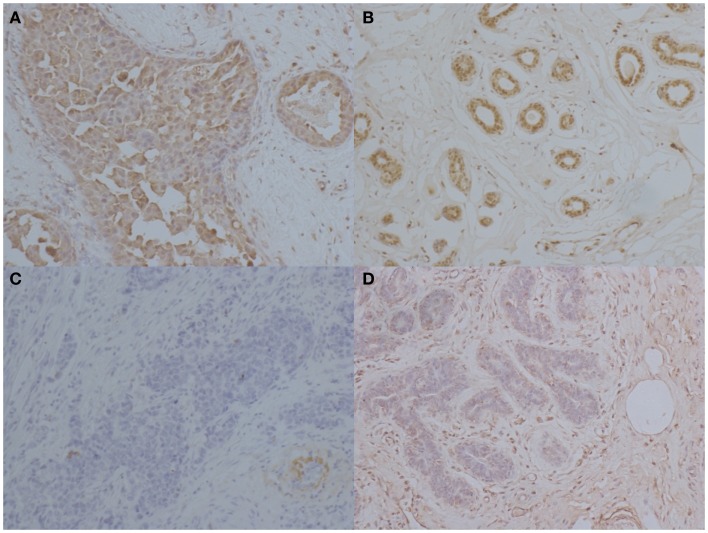
**Immunohistochemical staining of IL-33 in patients with breast cancers**. Representative images of immunohistochemical staining for IL-33 in breast carcinoma, adjacent tissue to tumor, and normal tissue. **(A)** High IL-33 expression in carcinoma tissues. **(B)** IL-33 expression in tissue adjacent to tumors. **(C)** Low IL-33 expression in carcinoma tissue. **(D)** IL-33 expression in normal breast tissue from BC patients (magnification: ×400).

Figure 2 shows a representative immunohistochemical staining of IL-33 in tissues from patients with BC. In 29 patients with BC, mean expression of IL-33 in carcinoma was 72.6% of cells in the tumor, which was significantly higher than in normal breast tissues from the same patients (p < 0.0001) as shown in Table 2. Interestingly, the mean expression of IL-33 in adjacent tissues to tumor was 64.1%, which was also significantly higher than in normal breast tissues from the same patients (*p* = 0.0002). However, the mean expression level of IL-33 was not statistically different between in cancer and adjacent tissues (p = 0.3561).

**Table 2:** **The percentage of IL-33-positive tissue in breast tumors, tumor-adjacent tissues, and normal tissues from breast cancer patients**.

Samples	IL-33 (%)			
	*N*	Mean	SD	P value^a^
Breast carcinoma tissue	29	72.6	34.5	<0.0001
Adjacent tissues to tumors	29	64.1	34.7	0.0002
Normal breast tissues from BC patients	25^b^	26.8	33.0	

*BC, breast cancer*.

*^a^Statistically significantly different was found between cancer tissues vs. normal breast tissues, and between adjacent tissues vs. normal breast tissues from BC patients, using unpaired Student’s t-test. No statistical significance was found between cancer tissues and adjacent tissues*.

*^b^In four cases, no normal breast tissues were available*.

### Clinicopathological analysis of cytoplasmic IL-33 expression in breast cancer tissues

Interleukin-33 was mainly detected in cytoplasm of BC cells as shown in Figure 2. With quantitative analysis, mean expressions of IL-33 were not significantly associated with the age at diagnosis, menopausal status, tumor size, AJCC stage, ER, PR, and Ki-67 expression (Table 3). In comparison with high grade tumors, low grade tumors showed significant higher IL-33 expression (p = 0.027). Interestingly, high IL-33 expression was more frequently observed in tumors with HER2 overexpression (p = 0.017). In patients with more than three lymph nodes involved, the expression of IL-33 was significantly higher than the patients with ≤3 metastases (p = 0.002). High IL-33 expression was also associated with family history of malignant tumor (p = 0.002).

**Table 3:** **Clinicopathological analysis of cytoplasmic IL-33 expression in breast carcinoma**.

Variables	IL-33 (%)			
	*N*	Mean	SD	P value
Age				
≤50 years	13	83.85	27.25	0.103
>50 years	16	63.44	37.71	
Menopausal status				
No	13	83.85	27.25	0.114
Yes	16	63.44	37.72	
Size
≤2 cm	13	68.85	32.28	0.607
>2 cm	16	75.63	36.87	
AJCC stage				
I + II	22	69.09	34.21	0.342
III	7	83.57	35.44	
Histological grade^a^				
1 + 2	15	87.33	18.70	0.027
3	11	58.64	41.96	
ER expression				
Negative	14	76.8	34.8	0.536
Positive	15	68.7	34.82	
PR expression				
Negative	16	73.44	34.09	0.887
Positive	13	71.54	36.25	
HER2 expression				
No	18	62.50	38.90	0.017
Yes	11	89.09	16.40	
Ki-67 expression				
Low	6	75.00	24.29	0.851
High	23	71.96	37.07	
Lymph node metastasis				
≤3	24	67.71	35.87	0.002
>3	5	96.00	8.94	
Family history				
No	26	69.81	35.34	0.002
Yes	3	96.67	5.77	

*^a^Two cases with invasive lobular carcinomas and one case with an intraductal papillary carcinoma*.

## Discussion

Interleukin-33, the newest member of IL-1 family, is a recently identified cytokine with diverse and context-dependent functions, and has been shown to bind to ST2. IL-33 has also been characterized as a potent inducer of T helper (Th) 2 immune responses, and is an important mediator for mucosal healing and epithelial restoration/repair (18). IL-33/ST2 axis can also promote Th1-type responses depending on the presence or absence of IL-12 (5). The influence of the IL-33/ST2 axis may be protective or pathogenic in various disease conditions, as it has a dual role in inflammatory disorders (8, 19). IL-33 plays a crucial role in inflammation and is associated with many diseases, such as giant cell arteritis (20), biliary atresia (21), and chronic obstructive lung disease (22). However, few data have been reported about the role of IL-33/ST2 axis in cancer, and little is known about the function of IL-33 in patients with BC.

In this study, we investigated the serum level and tissue expression of IL-33 in patients with BC. We found significantly higher serum levels of IL-33 in patients with BC, compared with patients with BBD, and higher expression of IL-33 in carcinomas and adjacent tissues to tumors, compared with normal breast tissue from the same patients.

The serum concentration of sST2, the soluble form of the receptor for both IL-33 and IL-1, has been shown to be elevated in patients with metastatic BC, and knockdown of the sST2 decreases ErbB2-induced cell motility in two different cell lines (23). However, there are no previous reports about IL-33 expression in serum or tissues of BC patients. In this study, serum levels of IL-33 are higher in patients with ER-positive tumors, predicting that the IL-33/ST2 axis may be involved in hormone receptor signaling. Moreover, in carcinoma tissues, IL-33 expression is significantly higher in HER2-overexpressing tissues, consistent with the report that its receptor sST2 is over-expressed to promote BC metastases upon ErbB2 activation in BC cell lines (23). In ST knock out mice models, lack of ST2 can suppress BC progression and metastasis, through enhanced cytotoxic activity of NK cells and increased systemic Th1/Th17 cytokines (24). Although we found no association between serum levels of IL-33 and in patients with more than three involved lymph nodes, the higher expression of IL-33 consistent with the IL-33/ST2 axis being involved in progression and metastasis of BC.

Of interest is that serum levels and carcinoma tissue expression of IL-33 are higher in patients with a family history of malignant breast carcinoma. As IL-33 may play an important role in immunosuppression of cancer for subsequent tumor progression and metastasis, and auto-immune diseases are usually hereditary, patients with a family history may be more likely to trigger or promote the process of immunosuppression (11). Whether and how IL-33 expression is linked auto-immune disease and familial cancers needs to be clarified.

Ki-67 is a cancer cell proliferation biomarker (25). IL-33 was higher in the low Ki-67 expression group, suggesting serum levels of IL-33 are negatively associated with BC proliferation. After analyzing cytokines associated with IL-33, only a decrease in IL-12 is observed in patients with BC, suggesting systemic IL-33 may not play an important role in BC immunity. In a murine model, the IL-33/ST2 axis has been demonstrated to facilitate intratumoral accumulation of immunosuppressive and innate lymphoid cells, and then promote BC growth and metastases (11). Similarly, in head and neck squamous cell carcinomas, administration of IL-33 promotes cancer cell migration and invasion through induction of epithelial-to-mesenchymal transition. Moreover, IL-33 has been shown to be a potential prognostic biomarker and target for new therapeutic strategies (26). Recent research suggests that in the breast tumor environment, tumor-infiltrating T lymphocytes (TILs) secrete IL-17A, to activate the MAPK pathway, promoting proliferation and resistance to conventional chemotherapeutic agents (27). The elevations of both IL-33 serum levels and immunohistochemical expression might promote BC progression and metastases through regulation of IL-12 pathway.

The local expression of IL-33 may be an important marker for differentiating malignant from normal/benign tissues. IL-33 expression in adjacent tissues also tends to be higher compared to normal tissues, suggesting that adjacent non-cancerous tissues may be similarly relevant to cancers in terms of anti-tumor immunity. Local IL-33 expression may also increase intratumoral accumulation of immunosuppressive lymphoid cells in patients with BC. However, in high grade tumor tissue, the expression of IL-33 is decreased compared to low grade tumor tissues, indicating that IL-33 may be more important in HER2-over-expressing tumors, and other cytokines may be involved in this crosstalk of regulation. Interestingly, IL-33 expression in serum and cancer tissues was contrary when comparing with ER and HER2 expression, although with statistical significance (Tables 1 and 3). Up to now, there were no reports about the relationship between IL-33 and ER or HER2. So we supposed that IL-33 may play different roles in system and in local tissues under different hormone conditions. The IL-33 may be involved in the resistence to endocrine therapy and Herceptin therapy of ER/HER2 positive patients with BC.

It is confirmed that IL-33 could activate, Th1, NK, NKT, and CD8^+^ T cells under certain pathophysiological conditions (11). On the other hand, IL-33 has a dual role in inflammatory disorders, anti- and pro-inflammatory. The tissue expression of IL-33 is significantly different, indicated that in carcinomas, immune cells may be recruited to anti-inflammatory and subsequent immunosuppression in HER2 overexpression tumors. In summary, this study indicated that serum IL-33 is higher in cancer patients compare to patients with BBD. Immunohistochemical staining demonstrated that IL-33 is higher in both cancerous and adjacent tissues compared to normal tissues, suggesting its role in BC progression and metastases. Thus, IL-33 may be a useful biomarker for prediction of malignant potential and immunosuppression of breast carcinomas.

## Author Contributions

Jing Liu and Guo-Jun Zhang conceived and designed the experiments; Jing Liu, Jia-Xin Shen, and Jia-Lin Hu performed the experiments; Jing Liu, Wen-He Huang, and Guo-Jun Zhang analyzed the data; Jing Liu, Jia-Xin Shen, Jia-Lin Hu, Wen-He Huang, and Guo-Jun Zhang contributed reagents and materials; Jing Liu, Jia-Xin Shen, and Guo-Jun Zhang wrote the paper.

## Conflict of Interest Statement

The authors declare that the research was conducted in the absence of any commercial or financial relationships that could be construed as a potential conflict of interest.
